# Convolutional neural network models describe the encoding subspace of local circuits in auditory cortex

**DOI:** 10.1101/2024.11.07.622384

**Published:** 2024-11-08

**Authors:** Jereme C. Wingert, Satyabrata Parida, Sam Norman-Haignere, Stephen V. David

**Affiliations:** 1 Behavioral and Systems Neuroscience Graduate Program, Oregon Health and Science University, Portland, OR 97239, USA; 2 Oregon Hearing Research Center, Oregon Health and Science University, Portland, OR 97239, USA; 3 Biostatistics and Computational Biology, University of Rochester, Rochester, NY 14642, USA

## Abstract

Auditory cortex encodes information about nonlinear combinations of spectro-temporal sound features. Convolutional neural networks (CNNs) provide an architecture for generalizable encoding models that can predict time-varying neural activity evoked by natural sounds with substantially greater accuracy than established models. However, the complexity of CNNs makes it difficult to discern the computational properties that support their improved performance. To address this limitation, we developed a method to visualize the tuning subspace captured by a CNN. Single-unit data was recorded using high channel-count microelectrode arrays from primary auditory cortex (A1) of awake, passively listening ferrets during presentation of a large natural sound set. A CNN was fit to the data, replicating approaches from previous work. To measure the tuning subspace, the dynamic spectrotemporal receptive field (dSTRF) was measured as the locally linear filter approximating the input-output relationship of the CNN at each stimulus timepoint. Principal component analysis was then used to reduce this very large set of filters to a smaller subspace, typically requiring 2–10 filters to account for 90% of dSTRF variance. The stimulus was projected into the subspace for each neuron, and a new model was fit using only the projected values. The subspace model was able to predict time-varying spike rate nearly as accurately as the full CNN. Sensory responses could be plotted in the subspace, providing a compact model visualization. This analysis revealed a diversity of nonlinear responses, consistent with contrast gain control and emergent invariance to spectrotemporal modulation phase. Within local populations, neurons formed a sparse representation by tiling the tuning subspace. Narrow spiking, putative inhibitory neurons showed distinct patterns of tuning that may reflect their position in the cortical circuit. These results demonstrate a conceptual link between CNN and subspace models and establish a framework for interpretation of deep learning-based models.

## Introduction

Auditory perception requires parsing and grouping statistical regularities in complex and highly variable sounds. Numerous spectro-temporal encoding models have been proposed to describe the computations performed by neurons in the auditory system. Models that successfully predict time-varying neural activity evoked by natural and other complex stimuli can be analyzed to determine what computations support accurate predictions. Since the advent of deep learning methods, application of these tools to the encoding model problem has shown that large convolutional neural networks (CNNs) and related artificial networks, provide powerful and generalizable auditory encoding models. As in other sensory systems (Yamins et al., 2014; Du et al., 2024), CNNs fit to LFP and single-unit neural data recorded from auditory cortex consistently perform better than classical linear-nonlinear spectro-temporal receptive field (LN) models and nonlinear variants of the LN model (Keshishian et al., 2020; Pennington and David, 2023).

While the high prediction accuracy of CNN-based encoding models has been demonstrated repeatedly, their complexity and high number of free parameters has made it difficult to determine what nonlinear computations account for their performance. One concern raised about deep learning models in general is that they may solve problems in ways that are not biological and thus their value to understanding biological computations may be limited (Feather et al., 2023). Being able to understand the key computations performed by a CNN can generate hypotheses about neural computations that can be tested directly against the biological network.

Sensory encoding is in its essence likely to involve complex nonlinear calculations, and any accurate encoding model must be commensurately complex. Subspace, or multi-filter, encoding models are a family of encoding models, derived from the LN modeling framework, that can perform complex computations but can also be visualized in terms of a relatively small number of spectro-temporal filters (Pillow and Simoncelli, 2006; Atencio et al., 2008; Meyer et al., 2017). This filter subspace can be easily visualized and defines the range of stimuli that modulate a neural signal. Among other computations, subspace models can account for neurophysiological phenomena, such as gain control, and logical computations, such as “and” gates, which cannot be captured by the LN model. While conceptually appealing and relatively easy to interpretable, subspace models have proven difficult to fit, especially with natural stimuli, which contain high-order correlations that present challenges to traditional model fitting algorithms. Thus, relatively few studies have successfully fit subspace models (Rust et al., 2005; Atencio et al., 2008), and published methods do not transfer readily to novel datasets (Pennington and David, 2023).

Here we test the hypothesis that a large, multi-layer CNN can be flattened into a low-dimensional encoding subspace. We used microelectrode arrays to record activity of multiple single units across cortical columns of primary and secondary auditory cortex of awake ferrets during presentation of a large and diverse natural sound stimulus set. After fitting CNN models to neural data, we developed a method for measuring neurons’ tuning subspace from the CNN. A new encoding model based on a low (3–10) dimensional subspace predicted neural activity nearly as accurately as the full CNN. Analysis of the subspace models revealed functional differences between neural populations within a cortical column, particularly among narrow-spiking, putative inhibitory neurons. Thus, subspace models derived from the CNN provided interpretable characterizations that reveal distinct nonlinear tuning properties across cortical populations.

## Results

### Local linear approximation to a convolutional neural network model defines a neuronal tuning subspace

Single-unit neural activity was recorded across layers of auditory cortex of awake, passively listening ferrets using linear microelectrode arrays (Du et al., 2011; Juavinett et al., 2019). Recordings were performed in 4 animals in primary (A1, 53 sites) and non-primary (PEG, 14 sites) auditory cortex (3–248 units/site, mean 47 units/site). Recordings were performed during the presentation of natural sound stimuli to characterize the sound encoding properties of each neuron. The performance of encoding models to is limited by the diversity of stimuli used to measure tuning (Wu et al., 2006). To maximize stimulus sampling diversity, sequences of brief natural sound segments (duration 50–800 ms, mean 150 ms) were presented in rapid succession with occasional silent gaps (0.2–2 sec). The sound segments were drawn from a large natural sound library and sampled to maximize the diversity of spectrotemporal patterns presented. A single experiment presented approximately 7000–63,000 unique sound segments (1000–9000 sec) for model fitting.

**Figure 1.**
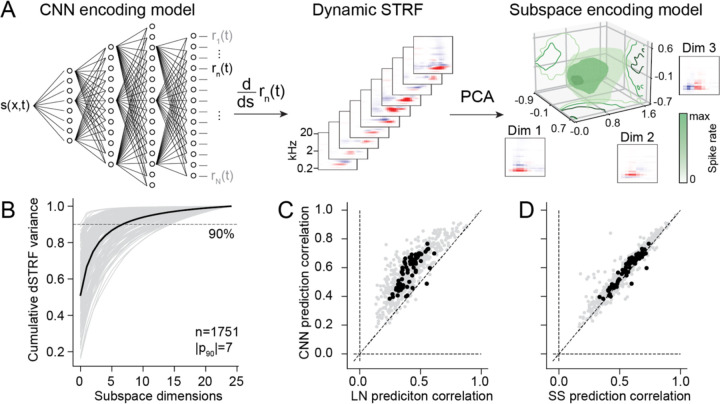
Mapping from convolutional neural network (CNN) to subspace encoding models. **A.** Population CNN model predicts time-varying neural activity recorded at a single site during presentation of a natural sound library. Dynamic STRF (dSTRF) is the locally linear approximation of the CNN model prediction for a single unit at each stimulus timepoint, computed by derivative of the relative to the stimulus spectrogram. The neuron’s tuning subspace is computed by principal components analysis (PCA) of the dSTRF. The subspace encoding model is then the mean response to the stimulus projected into the tuning subspace. **B.** Fraction dSTRF variance explained as a function of PCA count for each neuron (n=1751). Subspace model performance was optimal when dimensionality accounted for 90% of dSTRF variance (mean 7 dimensions). **C.** Scatter plot compares prediction correlation (noise-corrected correlation coefficient) between the LN versus CNN encoding models. Gray dots indicate performance of single units, and black dots show the median performance for each recording site (median 0.435 vs. 0.650, p<1.8e-12, Wilcoxon signed rank test). **D.** Scatter plot comparing subspace (SS) versus CNN model performance, plotted as in C (median 0.633 vs. 0.650, p=1.9e-10).

A four-layer convolutional neural network (CNN) was used to model the functional relationship between the stimulus spectrogram and the time-varying neural response (Fig. 1A, (Pennington and David, 2023)). To leverage statistical power for model fitting, a population model architecture was used, in which the first three model layers were shared across neurons within a recording site, and the final dense layer contained weights from the final shared layer to predict the activity of individual neurons (Pennington and David, 2023). The unique stimuli used for model fitting varied across recording sites. Model performance was evaluated using a held-out test dataset (6, 18-sec segments), which was fixed across all experiments and presented 10–20 times on trials interleaved with the fit stimuli. Consistent with previous studies, the CNN model was able to predict the time-varying response in the test set more accurately than a linear-nonlinear spectrotemporal receptive field (LN) model (Fig. 1C, 3B).

**Figure 2.**
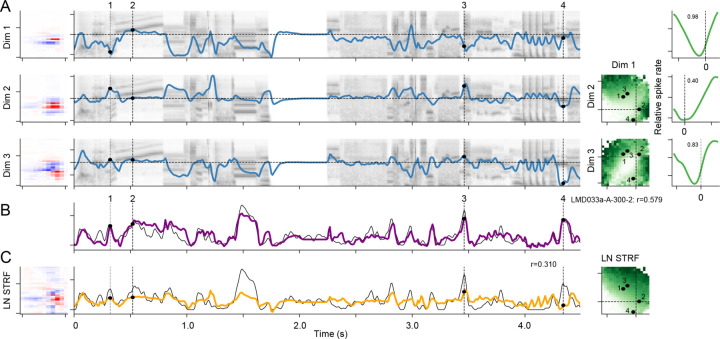
Example subspace model fit to a single A1 neuron. **A.** Subspace filters (left) are convolved with the stimulus spectrogram (second column, gray shading) to produce a projection into each dimension (3/7 significant filters shown). The projection defines a point in the tuning space, and the tuning surface indicates the predicted response at that moment in time (third column, green shading). In this example, the tuning spaces contain two distinct locations in the subspace that produce a strong response (upper left and lower right). Numbers indicate subspace positions at example points the time-varying projections (dashed vertical lines at left). Averages across the tuning surface show mean response as a function of the projection onto each tuning dimension (fourth column). In this case, the response functions are highly nonlinear, with strong responses for large positive and negative projections in each subspace dimension. **B.** Subspace model prediction (purple) overlaid with the actual peristimulus time histogram (PSTH) response (gray, r=0.579). **C.** LN model fit for the same neuron (left), LN model prediction (orange) overlaid with actual PSTH (middle, r=0.310), and average LN model prediction for stimulus projections onto the first two dimensions of the subspace model (right). LN tuning is constrained to lie in a plane, which cannot capture the nonlinear pattern observed in the subspace model in A.

While CNN-based auditory encoding models can account for neural response dynamics more accurately than traditional LN models (Fig. 1B), their complexity makes it difficult to understand what computations account for the increased predictive power. One way to visualize the functional properties of a CNN model is to measure the dynamic STRF (dSTRF), the derivative of the model response relative to the input stimulus at each point in time (Keshishian et al., 2020). This method is conceptually similar to saliency analysis sometimes used to understand deep learning models (Tjoa and Guan, 2020). dSTRF analysis produces a large collection of spectro-temporal filters, one per time sample (Figs. 1A, S1), which indicate the stimulus features modulating neural activity at each moment in time. dSTRFs for a single neuron often vary substantially over time but show some regular patterns in their structure. For a compact visualization of dSTRF variability, we performed principal components analysis (PCA) on the collection of dSTRFs for each neuron. For most A1 neurons, 2–11 principal components (mean 7) accounted for the majority of dSTRF variance (>90%), and 3 components accounted for a mean of 81% of dSTRF variance (Fig. 1B). The components measured for a single neuron often shared a best frequency but differed in spectral and/or temporal modulation tuning (Figs. 1A, 2).

Previous studies of sensory encoding have proposed that neural encoding is described by a subspace, or multi-filter, encoding model (Pillow and Simoncelli, 2006; Atencio et al., 2008; Meyer et al., 2017). In this family of models, each neuron is characterized by one or more linear filter, and sensory responses are predicted entirely from the projection of the stimulus into this low-dimensional subspace. In the case of one filter, the subspace model reduces to the LN model. We hypothesized that the collection of filters derived from the dSTRF define a neuron’s subspace. To visualize how the subspace accounts for tuning, we computed the average neural response across all stimuli at each point in the subspace (detailed examples in Figs. 2, S2). Subspace tuning curves show the average response to stimuli varying along a single subspace dimension (Fig. 2A, right column), and tuning surfaces show the average response to the projection onto a pair of dimensions (Fig 2A, second column from right). These tuning surfaces are typically complex and nonlinear. In contrast, predictions of the LN model projected into the same subspace reveals a planar tuning surface, consistent with a single tuning dimension captured by the LN model (Fig. 2B). Tuning subspaces tended have similar frequency and bandwidth tuning across neurons recorded in a single site, but the selectivity for spectro-temporal features did vary (Figs. S2, S3).

### Subspace encoding model is functionally equivalent to the CNN

The spectro-temporal tuning subspace provides a “flattened” neural network model. A CNN may contain many layers of many units, but the subspace tuning model describes the same computations as projection through a single layer of filters followed by a nonlinear combination of those filter projections. This tuning space can be used as a lookup table for the neural response to each stimulus, thus providing a compact visualization of the nonlinear computations that define a neuron’s encoding properties (Fig. 2).

**Figure 3.**
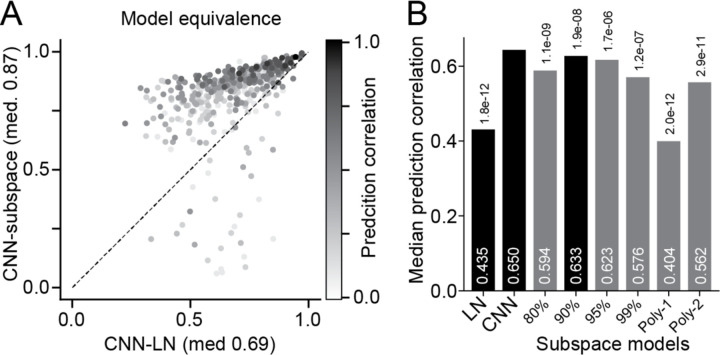
Functional equivalence of CNN and subspace encoding models. **A.** Scatter plot compares correlation coefficient between time-varying response predictions for CNN and LN models (x-axis) versus for CNN and subspace models (y-axis, n=1751). **B.** Median prediction accuracy for LN, CNN and subspace models. Subspace models varied in the number of PCA filters (80–99% of dSTRF variance). Polynomial models (right) constrained the mapping from subspace to prediction using a first- (Poly-1) or second order (Poly-2) polynomial. The Poly-1 model is equivalent to the LN model. For this dataset, prediction accuracy was maximal for the 90% model. All subspace models performed at least slightly worse than the full CNN model (Wilcoxon sign test, numbers at top indicate exact p-values, n=67 recording sites).

While the tuning subspace provides a compact description of neural sensory tuning, it is not immediately clear if it accounts for neural responses as well as the full CNN model. To determine how well the subspace accounts for time-varying neural responses, we fit a new model, where neural responses were predicted using only the projection onto the subspace filters accounting for 90% of dSTRF variance. A two-layer, densely connected network was used to fit the nonlinear tuning surface within the subspace. This subspace model was able predict neural responses with nearly the same accuracy as the full CNN (Fig. 1D). Moreover, a direct comparison of time-varying neural activity predicted by the two models showed strong correlation, indicating that the two models are functionally equivalent. Equivalence was substantially greater than between the CNN and LN models (Fig. 3A). Average model performance decreased slightly if filters accounting for more or less than 90% of dSTRF variance were included (Fig. 3B).

The tuning subspace describes the set of spectrotemporal patterns that influence a neuron’s activity, but it does not constrain the computations performed within that space. If the response is simply a linear combination of the projection of the stimulus into the subspace, the model reduces to the LN model. Indeed, a model constrained to use a linear combination of subspace projections performs similarly to the LN model (Poly-1, Fig 3B). Previous studies implementing spike-triggered covariance have modeled the interactions with a second-order polynomial (Pillow and Simoncelli, 2006; Kaardal et al., 2017). A second-order model in the tuning subspace performs better than the LN model, but worse than the full CNN or subspace model (Poly-2, Fig. 3B). Thus, while second-order interactions account for some of the subspace tuning, higher-order nonlinearities are required to fully describe the neural response.

### Neurons within a cortical column sparsely tile a local tuning subspace

Basic functional properties, such as best frequency and tuning bandwidth, differ between cell types and cortical depth in AC (Moore and Wehr, 2013; Li et al., 2015; Natan et al., 2017; Williamson and Polley, 2019). However, characterizations of spectrotemporal sound coding have been less conclusive, indicating, that variability is nearly as large within- as between groups (Eggermont, 1998; Atencio and Schreiner, 2010). We investigated whether the comprehensive tuning profile provided by the subspace encoding model revealed clearer distinctions between laminar and cell-type groups. Our electrode arrays were inserted perpendicular to the brain surface so that each experiment sampled activity across depths in the same cortical column. We used current source density and local field potential power profiles to determine depth based on standard references (Fig. S4A, (Lakatos et al., 2007; Mendoza-Halliday et al., 2024)). We also measured peak-to-trough width of each spike and observed a bimodal distribution, delineating a boundary of 0.35 ms between putative excitatory (regular spiking) and inhibitory (narrow spiking) neurons (Fig. S4B, (López Espejo and David, 2024)). Encoding model data was then assigned to four groups, superficial versus deep cortical layer (above or below the layer 3/4 boundary) and narrow versus regular spike width. We observed roughly the same model performance for the different neuron groups (Fig. S4C–D). Some differences in performance could be explained by differences in spike rate, which tended to be higher for narrow- than regular spiking neurons (Fig. S4E–F).

**Figure 4.**
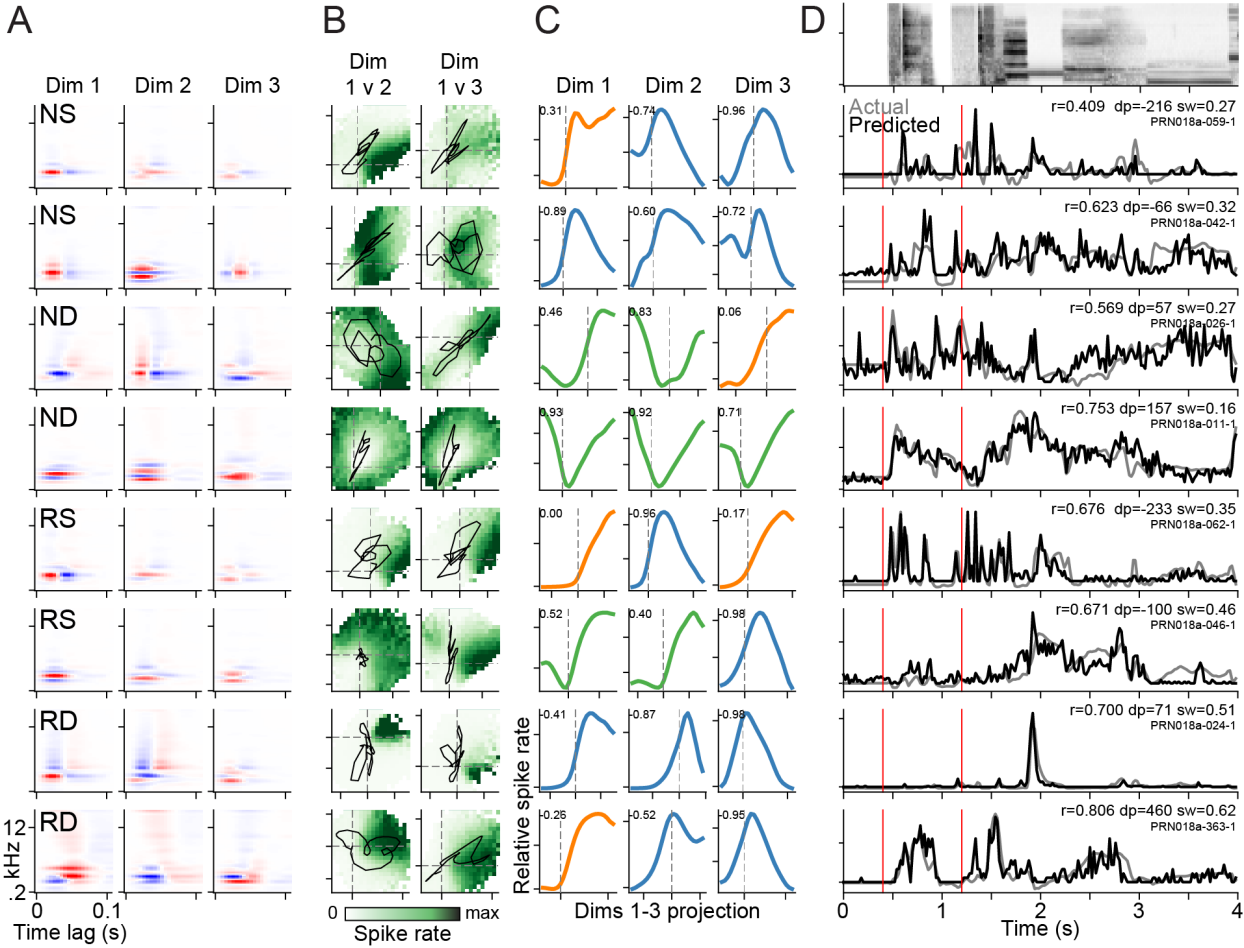
Diversity of subspace models within a single recording site. **A.** Three largest-variance subspace filters for eight example units, drawn from narrow-spiking superficial (NS, superficial to the layer 3/4 bourndary), narrow deep (ND), regular-spiking superficial (RS) and regular deep (RD) groups of neurons. **B.** Two-dimensional projections of the subspace response field for subspace dimensions 1 vs. 2 (left) and 1 vs. 3 (right). Darker green indicates higher spike rate. **C.** Marginal projections of the response field for each dimension. Color indicates type of tuning (orange: asymmetric, blue: symmetric suppressive, green: symmetric expansive), and numbers indicate tuning symmetry index (TSI). **D.** Top row shows 4-sec segment of spectrogram from the natural sound sequence used to test prediction accuracy. Each row below shows the predicted PSTH response (black) overlaid with the actual response (gray). Numbers indicate prediction correlation (*r*), microns below cortical layer 3/4 boundary (*dp*) and spike width in ms (*sw*).

Within a single recording site, we often recorded from multiple neurons in each of the four groups defined by recording depth and spike width (Fig. 4). Inspection of the subspace filters for different neurons showed broad similarity in tuning, such as in best frequency, but variability in the details of tuning to spectral and temporal modulation (Fig. 4A). Moreover, responses withing the tuning subspace varied quite substantially, producing very different PSTH responses between neurons recorded from the same site (Fig. 4B–D).

**Figure 5.**
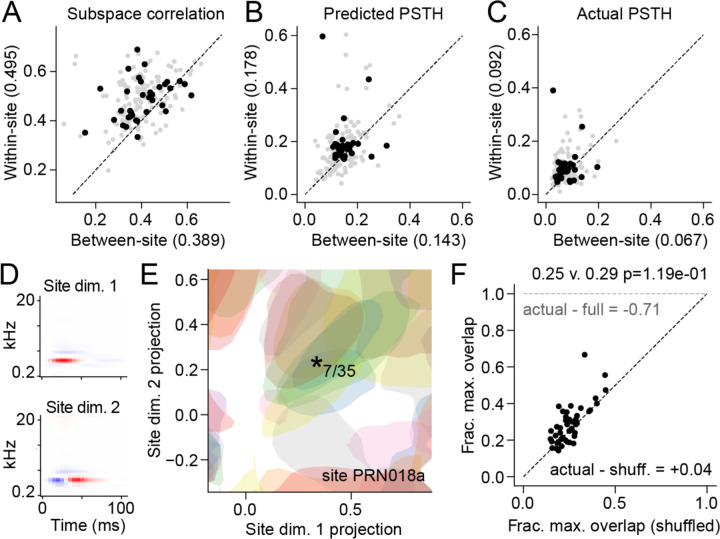
Similarity of tuning subspaces between units within- versus across recording sites. **A.** Scatter plot compares subspace similarity index (SSI) between pairs of units recorded from different sites (between-site) versus from the same site (within-site). Gray dots show individual pairs; black dots indicate mean for each recording site. SSI within-site is significantly greater than between-site (0.50 vs. 0.39, p=1.6e-4, sign test). To prevent bias from overfitting, comparisons were made only between two or more sites presented with identical stimuli, allowing fit of a single population model to data from multiple sites (n=39 sites). **B.** Scatter plot of correlation between predicted PSTH response (validation dataset) for pairs of units, plotted as in A. Correlation between predicted PSTHs was relatively low in all cases, but median correlation within-site was significantly greater than between-site (|0.18 vs. 0.14, p=3.2e-6, sign test). **C.** Scatter plot of correlation between actual PSTH response (validation dataset) for pairs of units, plotted as in A. Median correlation within-site was significantly greater than between-site (0.092 vs. |0.067, p=9.1e-16, sign test). **D.** Subspace dimensions for an entire recording site (Site dim.) were computed by applying PCA to dSTRFs pooled across all units in a recording site. Site dims. 1 and 2 are shown for 35 units from one example site (same site as examples in Fig. 4). **E.** Filled contour plot shows the area in the space defined by site dims. 1 & 2 for which each unit reaches >80% of maximum firing rate. The full set of 35 units tiles most of the subspace. Maximum overlap of units is 7/35 (*). **F.** Scatter plot of fraction maximum overlap in the site dim 1 & 2 subspace for shuffled versus actual 80% contours (n=39 sites with >10 sound-responsive units). Fraction of maximum overlap was not significantly difference from maximum overlap of the shuffled tuning spaces (p=0.12, sign test).

To assess the consistency of tuning within a recording site, we compared tuning subspaces between neurons within versus across recording sites. For each neuron, we determined the subspace filters that accounted for 80% of dSTRF variance. To account for the possibility that the number of filters associated with each neuron could vary, we defined a subspace similarity index (SSI) as the sum of correlation coefficients between each pair of filters, normalized by the smaller number of filters in the neuron pair. Thus, an SSI of 1 indicates complete overlap between two neurons, and 0 indicates no overlap (Fig. 5A). Because the populations CNN models were fit simultaneously for all neurons within a site, the shared model layers might bias SSI between neurons toward higher values. To control for this possibility, we focused on the subset of experiments in which the same natural sounds were presented during recordings from two or more recording sites (40 sites). A single model was fit using data pooled across the 2–3 sites, and SSI was computed within and across sites in that single model fit. The comparison of SSI for pairs of neurons within- versus across recording sites showed consistently greater similarity within a site (median 0.50 vs. 0.39, p=7.6e-5, sign test, Fig. 5A). Thus, neurons recorded from the same site tend to share a tuning subspace.

To gain a clearer understanding of shared encoding properties within a recording site, we measured the signal correlation (correlation coefficient) between the PSTH predicted by the subspace model and the actual PSTH for pairs of neurons within and across sites (Figs. 5B–C, respectively). Despite the relatively high SSI within site, signal correlation was substantially lower within recording sites, both for predicted and actual activity (median 0.18 and 0.092, respectively). Thus, despite spanning similar tuning spaces, pairs of neurons tended to produce very different time-varying activity.

We hypothesized that this difference between SSI and signal correlation could be explained by the overlap of nonlinear tuning within neuron’s subspaces. To explore this idea, we measured a single, shared subspace for all neurons recorded from the same site. This was accomplished by performing PCA across dSTRFs measured for all neurons in a recording site (Fig. 5D). We then computed the tuning surface for each neuron in this shared subspace, using the same approach as for the individual subspaces above. For a given site, then, we could plot the 80%-max response fields for each neuron in a common space. The response fields were often non-overlapping (Fig. 5E). Fields for individual neurons spanned a relatively small area, but the complete set tiled most of the subspace. As a result of this sparse tiling, the maximum number of overlapping fields tended to be relatively small. In an example site with 35 units, the maximum overlap was 7. When the position of the response field for each neuron was shuffled, the maximum overlap was 6 (Fig. S5). We observed a similar pattern across recording sites, where the average maximum overlap was only 4% greater than expected by chance, and 71% less than full overlap, which would be expected if all neurons shared the same response field in the tuning subspace (Fig. 5F). Thus, while neurons in a recording site tend to share a spectro-temporal tuning subspace, their responses are relatively uncorrelated, tiling the range of stimuli that span the tuning subspace.

### Local subspace overlap depends on neuronal cell type and cortical depth

**Figure 6.**
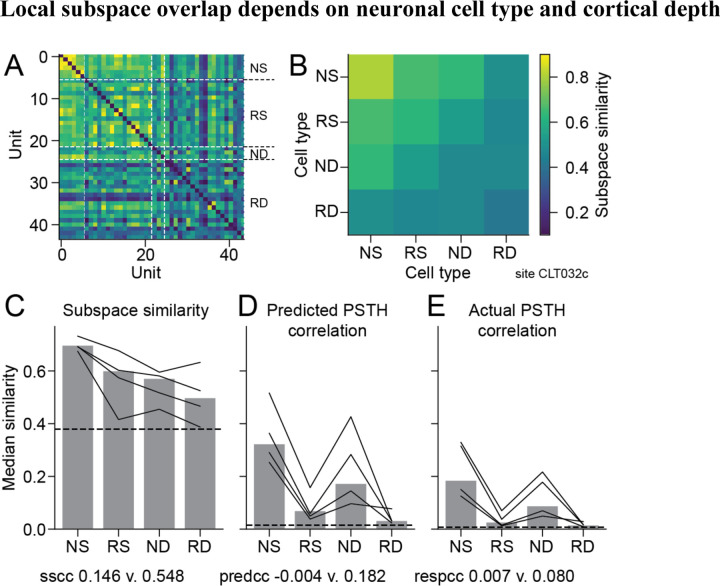
Subspace similarity within a local A1 population depends on neuronal cell type. **A.** Heatmap indicates subspace similarity index (SSI) between each pair of single units recorded from a single penetration, grouped by neuronal cell type (NS: narrow superficial, RS: regular superficial, ND: narrow deep, RD: regular deep). **B.** Heatmap shows mean SSI for the same site, averaged across cell types and plotted on the same color scale. SSI is highest for NS units. **C.** Bar plot shows SSI between pairs of neurons of each cell type, averaged across 51 A1 recording sites. Numbers indicate exact p-values for differences between groups (RS vs. NS: p=2.0e-4, NS vs. RS: p=0.033, RD vs. ND: p=0.60, Mann-Whitney U test). Lines indicate averages for data from each of the four animals studied. **D.** Signal correlation for predicted PSTH response between pairs of neurons in each cell type group, plotted as in C (RS vs. NS: p=1.6e-7, NS vs. RS: p=4.3e-3, RD vs. ND: p=2.5e-6, Mann-Whitney U test). **E.** Signal correlation for actual PSTH response, plotted as in C (RS vs. NS: p=3.6e-8, NS vs. RS: p=1.7e-4, RD vs. ND: p=7.7e-7, Mann-Whitney U test).

While neurons in a single recording site tend to span a similar tuning subspace, the question remained whether the degree of similarity depended on cortical depth or cell type. Using the four classifications defined above (NS: narrow superficial, RS: regular superficial, ND: narrow deep, RD: regular deep), we measured SSI for pairs of neurons from a single recording site within and across groups. Because narrow-spiking neurons are relatively rare, we focused this analysis on A1, where we collected most of our data. Inspection of SSI from a single recording suggested that narrow-spiking superficial (NS) units tended to have very similar tuning, while the subspace dimensions varied more widely across other units (Fig. 6A–B). We confirmed that this was the case across the entire dataset, with greater average SSI for pairs of NS units than any other pairs (Fig. 6C). The observation of greater SSI for NS units was consistent across each of the four animals studied (Fig. 6C, lines). We also compared signal correlation for predicted and actual PSTH between the same pairs of neurons (Figs. 6D–E). As above, signal correlation was generally low. However, it tended to be greater for narrow-spiking neurons, regardless of depth. Thus, we observe distinct patterns of subspace overlap and signal correlation for the different cell types. The high SSI and signal correlation for NS neurons indicates consistent tuning overall within this group. At the same time, the relatively high signal correlation for ND neurons is not corroborated by a correspondingly high SSI, suggesting that signal correlation is not determined entirely by subspace overlap.

### Diversity of nonlinear responses within the tuning subspace

Inspection of the subspace models shows a diversity of tuning nonlinearities across neurons (Fig. 4) that contribute to the tiling of responses in the tuning space (Fig. 5E). To quantify features of the tuning surface, a marginal tuning curve was calculated for each subspace dimension by projecting the stimulus into that single dimension and measuring the mean response for each value of that projection (Fig. 4C). Previous work has reported variation in the symmetry of subspace tuning curves (Homma et al., 2024). Consistent with these observations, tuning curves ranged from completely asymmetric (as would be expected for the linear-nonlinear model) to completely symmetric (consistent with as second-order or even-powered polynomial model, examples in Fig. 4C). Among symmetric tuning curves, the majority were downward facing, producing high firing range at intermediate input values and a decrease in firing rate at large positive and negative values. However, a minority of tuning curves were upward facing, producing high firing rate for large positive and negative inputs. The orientation of the symmetric tuning curves had distinct impact on the time-varying neural response. Neurons with downward-facing tuning curves tended to respond sparsely to a small number of stimuli (e.g., Fig 4, row 7), while neurons with upward facing tuning curves tended to respond to many stimuli (e.g., Fig. 4, row 4).

**Figure 7.**
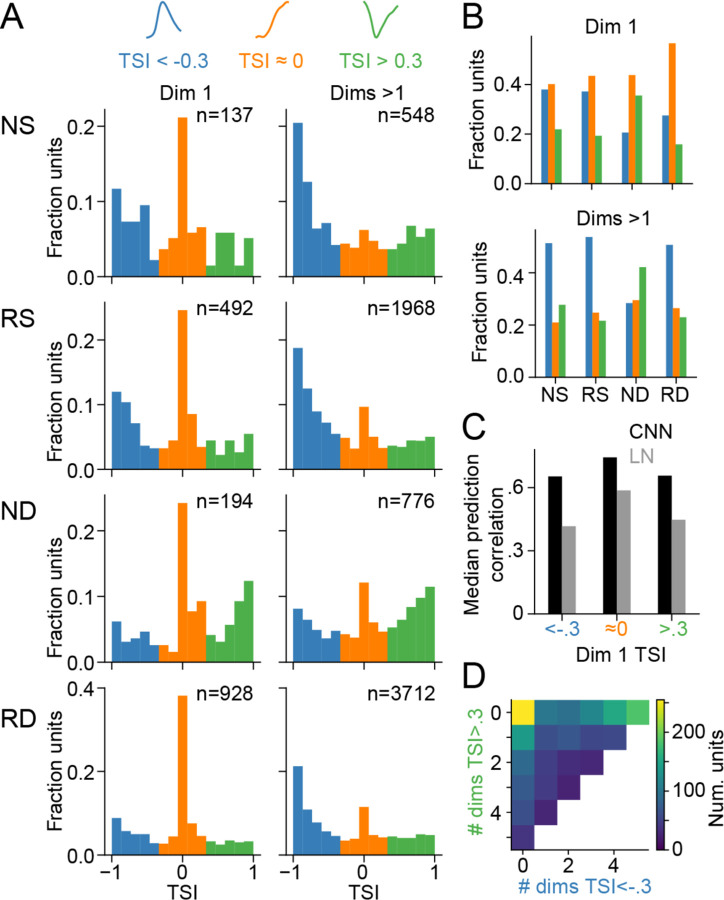
Diversity of subspace tuning nonlinearities across cell types. **A.** Histograms of tuning symmetry index (TSI) for subspace dimension 1 (first column) and dimensions > 1 (second column), grouped by cell type (rows). Color corresponds to TSI category (<−0.3 downward symmetric, >−0.3 & <0.3 asymmetric, >0.3 upward symmetric). **B.** Bars show fraction of TSI category for each cell type in subspace dimension 1 (top) and higher dimensions (bottom). **C.** Median prediction accuracy of the LN and CNN models grouped by the TSI category of the first subspace filter for each unit. **D.** Heatmap shows two-dimensional histogram of the number of significant expansive and suppressive nonlinearities for high-prediction accuracy A1 units.

To quantify both symmetry and the direction of the tuning curves, we defined a tuning symmetry index (TSI), computed as the difference between the summed derivative of the response curve above and below 0. A TSI of 1 or −1 indicates completely symmetric upward- or downward-facing tuning curve, respectively, and a value of 0 indicates a completely asymmetric tuning curve with a consistently positive or negative slope. Tuning curves were divided into terciles based on whether TSI was near −1, 0 or 1 (Fig. 7A). Consistent with previous work, and across all cell types, TSI for the first subspace dimension was more likely to have a value near 0 (Fig. 7A, left column, (Homma et al., 2024)). An asymmetric tuning curve should be captured more accurately by the LN model. Consistent with this prediction, the LN model tended to predict with greater accuracy for units with TSI near zero than for units with TSI near −1 or 1 (Fig. 7C). Units with TSI near −1 or 1 tended to show worse performance for the LN model and a greater improvement in prediction accuracy for the CNN model.

For higher subspace dimensions, TSI tended to be closer to 1 or −1, with fewer tuning curves in the asymmetric group (Fig. 7A, right column). Most often, TSI was negative, consistent with a decrease in response for large positive or negative stimulus projections onto that dimension. However, a minority of neurons had positive TSI values, indicating an increased response for large projection values. While the pattern was largely consistent across neuronal cell types, positive TSI values tended to occur much more frequently in the narrow-spiking deep neuron group (ND, Fig. 7B).

The sign of TSI was often similar for multiple subspace dimensions within a single neuron (see examples in Fig. 4). Thus, individual neurons tended to have either mostly positive or mostly negative TSI, with few showing representation of both (Fig. 7D). As seen in the examples, the spectro-temporal tuning properties of subspace dimensions also varied substantially across neurons and dimensions. While subspace filters were, by definition, orthogonal, they could have similar tuning properties. For example, of the two subspace filters had a quadrature phase relationship (either on the time or frequency axis), the net effect was to suppress (TSI<0) or enhance (TSI>0) responses to a stimulus based on its spectro-temporal modulation energy, independent of phase. To measure tuning properties independent of best frequency and response latency, we computed the modulation power spectrum of each subspace filter. We then averaged the modulation power spectra within each TSI and cell-type group. While patterns of modulation tuning varied substantially, subspace dimensions in the positive symmetric group (TSI near 1), tended to be tuned to rapid temporal modulations at or above 10 Hz (Fig. S6). If represented in quadrature phase, these filters may account for rate code (non-phase locked) responses to amplitude modulated stimuli (Liang et al., 2002).

## Discussion

We find that a low-dimensional tuning subspace can be readily extracted from a CNN-based sensory encoding model for neurons in auditory cortex (AC). A new encoding model based only on the projection of stimuli into the subspace performs nearly as well as the original CNN, indicating that this simpler and more interpretable model accounts for the key computations performed by the CNN. Analytically, the subspace model is closely related to multifilter models estimated using spike-triggered covariance (STC) and maximally informative dimensions (MID), thus establishing a conceptual link between CNNs and these earlier architectures (Pillow and Simoncelli, 2006; Atencio et al., 2008). While CNNs have been shown to account for sensory coding qualitatively better than other encoding models, understanding their key computations has been challenging (Yamins and Hong, 2014; Walker et al., 2019). We find that subspace models clearly describe nonlinear response properties and elucidate functional differences between neuronal cell types, identifying distinct functional properties of narrow-spiking, putative inhibitory neurons. While the current study focuses on subspace analysis of neurons in the auditory system, it is a general method that can be applied to neural network models for a wide range of neural systems.

### Diverse selectivity within a tuning subspace forms a sparse code in local cortical populations

As sensory information travels from the thalamus to cortex, the number of neurons available to represent a tonotopic input increases. This expansion of representation, which is consistent across sensory systems, has been proposed to support a sparse population code, in which individual neurons respond only to a small number of stimuli (Olshausen and Field, 1996; Willmore and King, 2009). A sparse representation can facilitate selection of sensory features for binding features into objects and selecting information to guide behavior. Analysis of subspace properties within local neural populations in auditory cortex revealed that nearby neurons share a similar subspace, but their tuning sparsely tiles that subspace. The variability in tuning nonlinearities across neurons produces diverse time-varying responses within a local population. This sparse tiling accounts for the relatively weak signal correlations within the local population, even though these neurons all share similar basic tuning properties, like best frequency and tuning bandwidth (Kanold et al., 2014). As a result, for any stimulus that falls within the tuning subspace, only a small fraction of neurons will produce responses. The degree of overlap was close to what would be expected if the response fields randomly tiled the tuning subspace. This minimal overlap suggests that recurrent connectivity within local circuits serves to carve out unique response fields for each neuron within a shared tuning subspace.

### Tuning within sensory subspaces accounts for gain control and sensory invariance

The shape of tuning nonlinearities imposes qualitative differences on neural response properties. Asymmetric nonlinearities produce a classic monotonic response, characteristic of the LN model. If the nonlinearity is symmetric a stimulus will have a similar effect on spiking whether it projects to a positive or negative value in the tuning subspace. For nonlinearities that are downward facing, large projections produce a decrease in firing. For neurons with multiple downward facing nonlinearities, this can produce gain control behavior. A neuron may response to a sound feature, but spectro-temporal energy anywhere in the downward facing dimensions will suppress that response. The fact that these dimensions are finite indicates that gain control effects are tuning rather than integrating non-selectively across sound features.

Tuning nonlinearities in a separate group are symmetric and upward facing. For these neurons, a feature projecting to large positive or negative values on the corresponding subspace dimension will produce an increase in firing. For a subspace filters in quadrature phase, this nonlinearity can have the effect of producing a phase-invariant response to a spectro-temporal feature, conceptually like complex cells in the visual cortex. In the case of modulations on the temporal axis, this computation can produce rate coding of temporally modulated sound (Liang et al., 2002). The same phase invariance can also occur on the spectral axis, as in the example in Fig. 2. This instantiation of phase invariance has not previously been reported for AC neurons, suggesting a new high-order computation performed by the auditory system.

### Functional equivalence of CNN-based and multi-filter encoding models

Understanding how the brain encodes and extracts information from dynamic natural sounds is a long-standing problem in sensory neuroscience. The classic linear-nonlinear spectro-temporal receptive field (LN STRF) describes encoding as convolution of the sound spectrogram with a linear spectro-temporal filter, followed by a static rectifying nonlinearity. Subspace encoding models have been proposed as a generalization of the LN STRF, in which the stimulus is convolved with two or more filters, and the response is then a nonlinear combination of the projection into this tuning subspace. Subspace models provide a logical, interpretable expansion of the LN STRF but have proven difficult to estimate accurately, especially for dynamic natural sounds. Subspace analysis provides a direct conceptual link between neural network-based encoding models and multi-filter encoding models previously proposed to describe encoding by the auditory and visual systems.

## Material and Methods

### Surgical procedures

All procedures were approved by and performed in accordance with the Oregon Health & Science University Institutional Animal Care and Use Committee (IACUC) and conform to the standards of the Association for Assessment and Accreditation of Laboratory Animal Care (AAALAC) and the United States Department of Agriculture (USDA). Four neutered young adult ferrets (3 male, 1 female) were obtained from a supplier (Marshall Farms). In each animal, sterile head-post implantation surgeries were performed under anesthesia to expose the skull over the auditory cortex (AC) and permit head-fixation during neurophysiology recordings. Surgeries were performed as previously described (Slee and David, 2015; Saderi et al., 2020; Heller et al., 2023). After removing tissue and cleaning the skull, two stainless steel head posts were anchored along the midline using light-cured bone cement (Charisma, Kulzer). To improve implant stability, 8–10 stainless self-tapping set screws were mounted in the skull. Layers of bone cement were used to build the implant to a final shape amenable to neurophysiology and wound margin care, which included frequent cleaning and sterile bandaging. Following a two-week recovery period, animals were habituated to head-fixation and auditory stimulation.

### Acoustic stimuli

Stimuli for model fitting consisted of large sequences of natural sound segments. Segments were drawn from one of two large corpuses: Audioset or Pro Sound Effects (PSE) (Core 3 Complete). The Audioset corpus is comprised of recordings from over 2 million YouTube videos. The PSE corpus is comprised of 371,089 high-quality recordings and sound effects (over 3,000 hours of audio). We selected 484,375 segments from each corpus (250k 50-ms segments, 125k 110-ms segments, 62.6k 190-ms segments, etc.). Five segment durations were used (50, 110, 190, 420, and 780 ms). The pseudo-logarithmic distribution of durations avoided exact integer multiples to minimize the likelihood of harmonic ringing in the neural responses. Each sequence was 17.79 seconds in duration and included 124 segments (64 50-ms segments, 32 110-ms segments, 16 190-ms segments, etc.) plus 5 segments of silence, one per duration. The ordering of segments was random and the sound level of segments was sampled from a uniform distribution with a 20 dB range. Segments were combined using crossfading at the boundary to avoid click artifacts (10 ms Hanning window).

Segments were selected by randomly sampling audio files without replacement from the Audioset and PSE corpus. To avoid selecting highly similar segments, an auditory excitation pattern was computed for each segment, and segments were selected to have distinct excitation patterns. The excitation pattern was computed by multiplying the power spectrum $\left({\left|FFT\left(x\right)\right|}^{2}\right)$ of the segment with cosine filters designed to coarsely mimic cochlear filtering in the ferret, followed by compression (raising the outputs to the 0.3 power). The structure of the cosine filters has been described previously for humans (McDermott and Simoncelli, 2011). The only difference is that we used an alternate formula for calculating $ERB_{N}$ derived based on (Sumner and Palmer, 2012):

$$\frac{1}{0.31*\left(1-0.533\right)}f^{\left(1-0.533\right)}$$

We excluded segments whose excitation pattern was similar to a previously selected segment from the same recording (r > 0.8) or whose whose excitation pattern was similar (r > 0.8) to more than 5% of previously selected segments (computed by randomly sampling up 10,000 previously selected segments). Audioset contains a large amount of speech and music. To prevent these stimuli from dominating the stimulus set, we prevented any one of the category labels from the Audioset ontology from comprising more than 20% of the selected Audioset segments. Collectively, these criteria caused us to reject 46% of the randomly sampled files from PSE and 82% of randomly sampled segements from Audioset (the larger number for Audioset reflects the 20% category maximum for those stimuli).

For computational speed, we only excerpted segments from the first 10 seconds of each sound file (some PSE files are much longer than this duration). Stereo sounds were converted to mono by selecting the left channel, and all stimuli were resampled to 44100 Hz. For each file, we divided the recording into frames of the desired segment duration (hop equal to half the segment duration). Segment durations were 40 ms longer than those noted above to allow for crossfading. We computed the power of each frame and discarded any frames whose power was 25 dB below the maximum power across all frames in that recording (to discard silent periods). We also discarded frames that violated the excitation pattern similarity criteria described above. We then selected the segment with the greatest power from those remaining.

Stimuli for model testing consisted in part of 4 sequences created using the same approach described above. In addition, the test set contained 2, 18-second sequences composed of 1-second segments of natural and synthetic sounds tested in prior experiments from our lab (Pennington and David, 2023). These included 6 synthetic segments comprised of tones and noises and 18 natural sound segments that included ferret vocalizations, marmoset calls, speech, music, and environmental sounds. Each sequence was composed of 8 segments with a 500-ms buffer period between each segment. Thus, in total, the test set consisted of 6 sequences with a total duration of ~100 seconds (4*17.79+2*18=107.16). These sequences were presented between 10 and 20 times depending on the amount of recording time available.

Digital acoustic signals were transformed to analog (National Instruments), amplified (Crown), and delivered through a free-field speaker (Manger) placed 80 cm from the animal’s head, 0° elevation, and 30° contralateral to the recording hemisphere. Stimulation was controlled using custom MATLAB software (https://bitbucket.org/lbhb/baphy) and all experiments took place inside a custom double-walled sound-isolating chamber (Professional Model, Gretch-Ken). Each stimulus sequence was presented at 65 dB SPL (RMS power).

### Neurophysiology

Neurophysiological recordings were performed in awake, passively listening animals while head-fixed. To prepare for neurophysiological recordings, a small craniotomy (0.5–1mm) was opened over AC. Recording sites were targeted based on tonotopic maps and superficial skull landmarks (Bizley et al., 2005; Atiani et al., 2014) identified during implantation surgery. Initially, tungsten microelectrodes (FHC, 1–5MΩ) were inserted into the craniotomy to characterize tuning and response latency. Short latency responses and tonotopically organized frequency selectivity across multiple penetrations defined the location of primary auditory cortex (A1) (Bizley et al., 2005), whereas secondary auditory cortex (posterior ectosylvian gyrus, PEG) was identified as the field ventrolateral to A1. The border between A1 and PEG was identified from the low-frequency reversal of the tonotopic gradient.

Once a cortical map was established, subsequent single-unit recordings were performed using two different electrode configurations. Experiments in animal 1 recorded neural activity using acute insertion of 64-channel silicon electrode arrays, which spanned 1.05mm of cortical depth (Du et al., 2011). Experiments in animals 2–4 recorded activity using Neuropixels short NHP probes (IMEC, 384/960 selectable channels, (Jun et al., 2017)). Probe tips were sharpened in lab or purchased pre-sharpened to permit penetration of dura. Neuropixels probes were implanted semi-chronically using a manual microdrive (modified from (Vöröslakos et al., 2021)) secured on the animal’s implant with dental cement. The array was implanted and allowed to settle for at least 24 hours to permit stable recordings. After recordings were complete at one site, the probe was explanted, sterilized, and reimplanted in a new craniotomy. Typically, about 150 of the 384 active channels spanned the depth of AC, as determined by current source density analysis (see below). Electrophysiological signals were amplified (RHD 128-channel headstage, Intan Technologies, or Neuropixels headstage, IMEC), digitized at 30 KHz (Open Ephys) (Siegle et al., 2017), and saved to disk for further analysis.

Spikes were sorted offline using Kilosort2 (https://github.com/MouseLand/Kilosort2) (Pachitariu et al., 2016), and sorting results were manually curated in phy (https://github.com/cortex-lab/phy). A contamination percentage was computed by measuring the cluster isolation for each sorted and curated spike cluster, which was classified as a single unit if contamination percentage was less than or equal to 5%. Clusters with contamination above 5% were classified as multi-unit and excluded from analysis.

### Laminar depth analysis

We used current source density analysis to classify units by cortical layer (1/3, supragranular; 4, granular; 5/6 infragranular). The local field potential (LFP) signal was generated by lowpass filtering either the raw signal from the 64-channel silicon probe or the LFP signal from the Neuropixel probe below 250Hz using a zero-phase shift (filter-filter method) 4^th^ order Butterworth filter, followed by down-sampling to 500Hz. A custom graphical interface was used to mark boundaries between layers, based on features of average sound-evoked LFP traces sorted by electrode depth (https://github.com/LBHB/laminar_tools). Layer-specific features included the pattern of current source density (CSD) sinks and sources evoked by best frequency-centered broadband noise bursts. Patterns were selected to match auditory evoked CSD patterns seen in AC of multiple species (Maier et al., 2010; Schaefer et al., 2015; Davis et al., 2023; Mendoza-Halliday et al., 2024). Each unit was assigned a layer based on the boundaries above and below the channel where its spike had largest amplitude.

### Spike width classification

We classified neurons as narrow- and broad-spiking based on the average width of the waveform. Width was calculated as the time between the depolarization trough and the hyperpolization peak (Trainito et al., 2019). The distribution of spike width across neurons was bimodal, and the categorization threshold was defined as the minimum between the bimodal peaks. Filtering properties differed between 64-channel probes and Neuropixels, thus categorization threshold was defined as 0.35 ms and 0.375 ms, respectively. Consistent with studies in other systems, recordings from optogenetically labeled GABAergic neurons in ferret auditory cortex have demonstrated that these inhibitory neurons fall entirely in the narrow-spiking group (López Espejo and David, 2024).

### Convolutional neural network (CNN) encoding model

Encoding model analysis was used to describe the transformation from stimulus spectrogram to neural response. The sound-evoked response was defined as the peristimulus time histogram (PSTH),$\;r\left(t\right)$, a neuron’s time-varying spike rate, sampled at 100 Hz (10 ms bins). Model input was the spectrogram, $s\left(f,t\right)$, of the sound waveform, computed using a second-order gammatone filter bank and log compression to account for the action of the cochlea (Lyon and Mead, 1988). The spectrogram was fixed for each model rather than fitting the spectrogram’s parameters, since we have observed little benefit on model performance from this additional complexity. The filter bank consisted of *F* = 32 filters with $f_{j}$ spaced logarithmically from $f_{\text{low}}=\;200$ to $f_{\text{high}}=\;20,000\;$ Hz (approximately 1/6 octave per bin). Filter bank output was smoothed and downsampled to 100 Hz to match the sampling of the neural PSTH. For the stimulus spectrogram, defined as a function of sound frequency and time, $s\left(f,t\right)$, a causal encoding model prediction at time, t, for neuron, i, is then a function of the stimulus up until that time, $r_{i}\left(t\right)=H_{i}\left[S\left(t\right)\right]$, where $S\left(t\right)=\left[s\left(f,1\right)\ldots s\left(f,t\right)\right]$.

#### Linear-nonlinear model.

The linear-nonlinear spectro-temporal receptive field (LN) model is widely used in studies of neural auditory coding (Eggermont et al., 1983; Theunissen et al., 2001; Calabrese et al., 2011) and was used as a baseline reference for this study (Fig. 1). To leverage statistical power in the neural population data and permit direct comparison with the population CNN model, below, the LN model was implemented using a shared filter space across neurons. The population LN model is analytically identical to the traditional single-neuron LN model but can predict with slightly greater accuracy for neurons with low response reliability (Pennington and David, 2023). The first stage of the population LN model convolves a bank of M finite impulse response (FIR) filters, h, with the stimulus spectrogram:

(1) $$r_{m}\left(t\right)=\sum_{f}^{F}\sum_{u}^{U}h_{m}\left(\text{f,u}\right)s\left(f,t-u\right)$$

Here, we used M=100 filters, each containing F= 32 spectral channels and U= 25 temporal bins. Filters are implemented with rank-1 factorization. Higher rank filters could be used, but we have found that in a trade-off between larger filter bank (greater N) and higher-rank filters, the additional filters provide better model performance (Thorson et al., 2015). Activity in this filtered space is then linearly weights to generate a linear prediction for each neuron i:

(2) $$r_{lin,i}\left(t\right)=\sum_{m}^{M}w_{i}\left(m\right)\;r_{m}\left(t\right)$$

Finally, a static sigmoid nonlinearity that mimics spike threshold and firing rate saturation is applied to the result of this convolution to produce the final model prediction. For this study, we used a double exponential nonlinearity:

(3) $${\text{r}}_{i}\left(t\right)={\text{b}}_{i}+{\text{a}}_{i}\;\;\exp\left[-\exp\left(k_{i}\left(r_{\text{lin},\text{i}}\left(t\right)-s_{i}\right)\right.\right]$$

where the baseline spike rate, saturated firing rate, firing threshold, and gain are represented by b, a, s and k, respectively (Thorson et al., 2015).

#### Population CNN model.

The CNN model employed an architecture developed in a previous study of natural sound encoding in AC (Pennington and David, 2023). Like the LN model, this architecture implemented a subspace shared across neurons, and the time-varying activity of individual neurons was computed in the last layer, as the weighted sum of outputs from the penultimate (shared) model layer (Walker et al., 2019; Pennington and David, 2023). The initial two layers were composed of 1D linear convolutional filters that performed a convolution in time and weighted sum across input channel (layer 1: 80 32×15 linear filters, layer 2: 100 80×10 linear filters, Eq. 1). This was followed by two densely connected layers (layer 3: 100×100, layer 4: 100x*N*), where N was the number of units being fit. Each layer was separated by ReLU activation functions. The final dense layer was followed by a double exponential nonlinearity, identical that that used in in the LN model (Eq. 3) and fit individually for each neuron.

### Model optimization

All fitting was performed exclusively with the estimation dataset, and the validation set was used only for measuring prediction accuracy. Prior to fitting, all input (stimulus) and output (time-varying spike rate) channels were normalized to a range of 0 to 1. Model parameters were then fit using TensorFlow’s implementation of the Adam algorithm for stochastic gradient descent, using a mean squared error (MSE) loss function, with L2 regularization on the linear weight parameters (Abadi et al., 2016). Loss was computed for all neurons in the dataset simultaneously. To obtain an initial coarse fit, the final double exponential nonlinearity was replaced with a simple level shift, and the fit was performed with a relatively high learning rate (0.01) and stop tolerance (0.001). The double exponential was then restored and fitting continued with smaller learning rate (0.001) and tolerance (10^−4^). To mitigate overfitting, an 8-fold jackknifing procedure was used, in which a distinct 12.5% of the fit set was excluded from the fit data set on each iteration. Each jackknife was initialized and fit starting at 5 random initial conditions. The highest performing model for each jackknife was saved for subsequent analysis.

### Tuning subspace analysis

#### Dynamic spectro-temporal receptive field (dSTRF) measurement.

While the population CNN model describes a complex nonlinear function, the stimulus-response relationship at each time point during stimulation can be approximated as a linear spectro-temporal receptive field, termed the dSTRF _. This locally linear approximation can be measured as the derivative of the response predicted by the CNN model at each timepoint, ${\text{r}}_{i}\left(t\right)={\text{H}}_{i}\left[S\left(f,0\ldots t\right)\right]$, relative to the input stimulus,

(4) $$d_{i,t}\left(f,u\right)=\frac{d}{d\;S\left(f,t-u\right)}{\text{H}}_{i}\left[S\left(f,0\ldots t\right)\right]$$

Intuitively, this calculation corresponds to fitting an LN model using only small perturbations in the stimulus at each frequency and time lag, $\delta \left(f,t-u\right)$. The dSTRF was measured with a maximum time lag, U=250ms, which corresponds to the maximum integration time of the population CNN. In practice, were measured the dSTRF by computing Jacobian of the population CNN output at each stimulus timepoint, which is equivalent to Eq. 4|. To reduce noise, the dSTRF was measured using each of the 8 jackknife model estimates. A shrinkage function was used to attenuate noise in the average across jackknifes oem(Brillinger, 1996). Thus, for a single neuron and stimulus of length T, the final dSTRF consisted of T linear spectro-temporal filters, $d_{i,t}\left(f,u\right)$.

#### Subspace encoding model.

The large collection of dSTRFs measured for each neuron was projected into a low dimensional space by principal component analysis (PCA). The frequency, f, and time lag, u, dimensions of the dSTRF were stretched into a single axis, and PCA was performed along the time axis, t. A low-dimensional subspace was then defined by the N components explaining the most variance in the dSTRF, $g_{i,j}\left(f,u\right)$, j=1…N. For most analysis, we chose N to account for 90% of variance, but different threshold values were assessed in some analyses. For subsequent analysis, the stimulus was projected into this low dimensional subspace,

(5) $$x_{j}\left(t\right)=\sum_{f}^{F}\sum_{u}^{U}g_{i,j}\left(\text{f,u}\right)s\left(f,t-u\right),$$

Nonlinear tuning in the subspace was visualized computing the mean actual or predicted firing rate at each point in that space. To determine how well the tuning subspace accounted for sensory responses, a densely connected two-layer neural network was used to model the relationship between the subspace projection and the time-varying neural activity in the fit data,

(6) $$\text{r}\left(\text{t}\right)=\text{F}\left(x_{1}\left(t\right),x_{2}\left(t\right),\ldots ,x_{N}\left(t\right)\right)$$

This model could account for arbitrary nonlinear interactions within the subspace but could not account for any spectrotemporal tuning outside of the *N* subspace dimensions.

#### Second-order subspace model.

Multi-filter models using spike-triggered covariance use a second order model to define the relationship between a subspace representation and the neural response. To implement this architecture, we used a second order model to fit the mapping between stimulus subspace and time-varying response. For the N-dimensional subspace stimulus, $x_{1}\left(t\right)\ldots x_{\text{N}}\left(t\right)$, the second order model response is:

(7) $$r\left(t\right)=\sum_{i,j}^{N}w_{ij}x_{i}\left(t\right)x_{j}\left(t\right)+\sum_{i}^{N}v_{i}x_{i}\left(t\right)+r_{0}$$

Second-order coefficients, $w_{ij}$, are weights applied to products of values in the subspace channels i and j. When i=j, a positive value indicates an increase in response when the projection on that dimension has either a large positive or negative value, and a negative value indicates a decreased response.

#### Subspace similarity index (SSI).

To measure the similarity of tuning subspaces between a pair of neurons, i and j, we computed the sum of the correlation coefficient between each pair of filters and normalized by the number of filters.


(8) $$\text{SS}{\text{I}}_{i,j}=\sum_{m,n}^{M,N}cc\left[g_{i,m}\left(\text{f,u}\right),g_{j,n}\left(\text{f,u}\right)\right]/\min\left(M,N\right)$$

For the current study, we measured SSI between the M=N=4 largest filters from each neuron.

#### Tuning symmetry index (TSI).

To characterize the shape of the marginal tuning curve for a single subspace dimension, TSI distinguished between symmetric downward facing (values near −1), symmetric upward facing (values near 1) and asymmetric (values near 0). For tuning curve, $y\left(x\right)$, with derivative, $y^{\prime }\left(x\right)=dy/dx$,

(9) $$\text{TSI}=1-|\sum_{x}^{⬚}y^{\prime }|/\sum_{x}^{⬚}\left|y^{\prime }\right|,\text{if}\sum_{x>0}^{⬚}y^{\prime }-\sum_{x<0}^{⬚}y^{\prime }>0\;\;\;\;\;\;\;\;\;=-1+\left|\sum_{x}^{⬚}y^{\prime }\right|/\sum_{x}^{⬚}|y^{\prime }|,\text{otherwise}$$

The derivative was approximated by discretizing the tuning curve to have 20 bins spanning 99% of the values in x spanned by the stimulus projection onto that dimension.

### Statistical analysis

For all pairwise statistical tests we performed a Wilcoxon signed-rank test (sign test). For statistical tests for unpaired or across area comparisons we used a Mann-Whitney U test. Significance was determined at the alpha = 0.05 level. The number of neuron/sound pair combinations and animals for each comparison are listed in the main text or figure legends, as are exact p-values.

## Supplementary Material

Supplement 1

## Significance statement

Auditory cortex mediates the representation and discrimination of complex sound features. Many models have been proposed for cortical sound encoding, varying in their generality, interpretability, and ease of fitting. It has been difficult to determine if/what different functional properties are captured by different models. This study shows that two families of encoding models, convolutional neural networks (CNNs) and tuning subspace models account for the same functional properties, providing an important analytical link between accurate models that are easy to fit (CNNs) and models that are straightforward to interpret (tuning subspace).
