# Supplementary material for: Convolutional neural network models describe the encoding subspace of local circuits in auditory cortex
Source: bioRxiv. 2024 Nov 8:2024.11.07.622384. Preprint. [Version 1] doi: 10.1101/2024.11.07.622384 (PMC11581007; doi:10.1101/2024.11.07.622384)
Supplement: Supplement 1 [file media-1.pdf]

Supplemental Figures

Convolutional neural network models describe the encoding subspace of local circuits in auditory cortex

Jereme C. Wingert[1,2], Satyabrata Parida[2], Sam Norman-Haignere[3], Stephen V. David[2,*]
[1] Behavioral and Systems Neuroscience Graduate Program, Oregon Health and Science University,
[2] Oregon Hearing Research Center, Oregon Health and Science University, Portland, OR 97239, USA
Portland, OR 97239, USA
[3] Biostatistics and Computational Biology, University of Rochester, Rochester, NY 14642, USA
Portland, OR 97239, USA
* Correspondence: davids@ohsu.edu (S.V.D.)

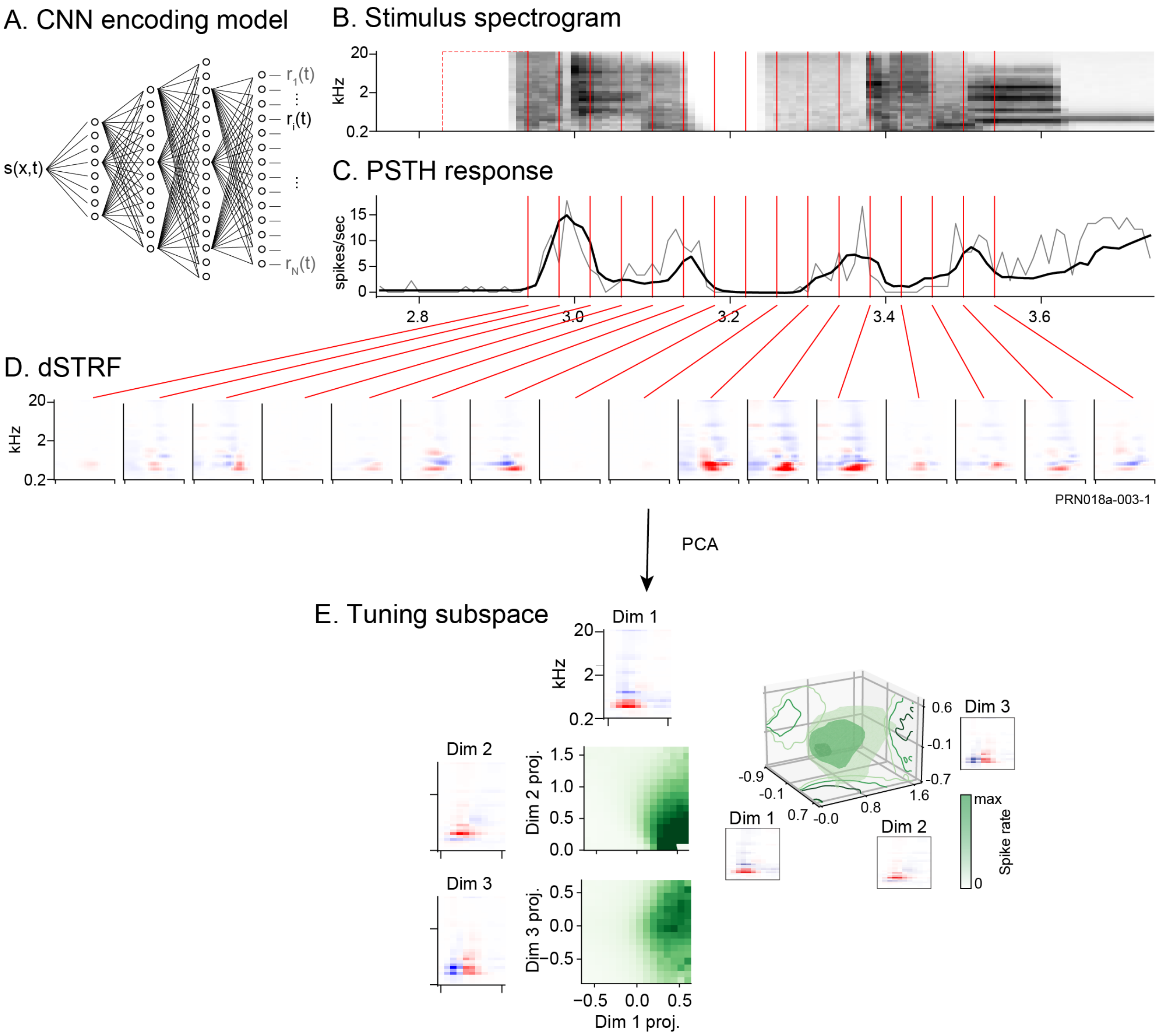

Figure S1. A. Natural sound encoding by single unit neurons recorded from ferret auditory cortex was modeled using a population convolutional neural network (CNN). **B.** Spectrogram of natural sound sequence presented during one experiment. **C.** Time-varying peri-stimulus time histogram (PSTH) response recorded from one neuron during presentation of stimulus in B (gray). PSTH predicted by the CNN model overlaid in black. **D.** Dynamic spectro-temporal receptive field (dSTRF) is calculated as the derivative of the model output relative to the stimulus input. Panels show dSTRF calculated for the unit in B at example time points (vertical bars in C). **E.** PCA is applied to the large collection of dSTRFs to compute a small number of spectrotemporal filters that define the tuning subspace, i.e., the spectro-temporal domain of stimuli that influence neural activity. The subspace encoding model is the nonlinear function that predicts neural activity from the stimulus projection into the subspace. Heatmaps at left show response rate as a function of projection onto each of the three largest subspace dimensions (green indicates higher spike rate). Heatmap at right shows 3D representation of tuning subspace.

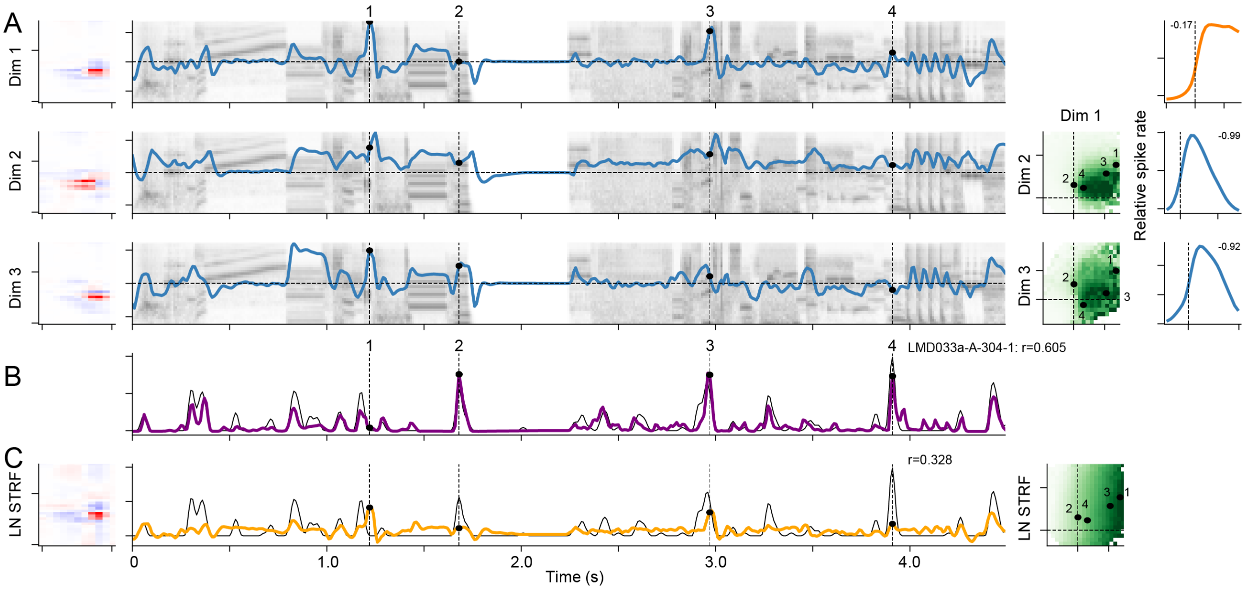

Figure S2. Detailed example of subspace encoding model, plotted at in Fig. 2. **A.** Subspace filters (left) are convolved with the stimulus spectrogram (second column, gray shading) to produce a projection into each dimension (3/7 significant filters shown). Tuning surface indicates the predicted response at that moment in time (third column, green shading). Numbers indicate subspace positions at example points the time-varying projections (dashed vertical lines at left). Averages across the tuning surface show mean response as a function of the projection onto each tuning dimension (fourth column). For this neuron, the first dimension has an asymmetric, monotonic nonlinearity, and dimensions 2 and 3 have a symmetric, suppressive nonlinearity. **B.** Subspace model prediction (purple) overlaid with the actual peri-stimulus time histogram (PSTH) response (gray, r=0.605). **C.** LN model fit for the same neuron (left), LN model prediction (orange) overlaid with actual PSTH (middle, r=0.328), and average LN model prediction for Dim 1 vs. Dim 2 projections in the subspace model (right).

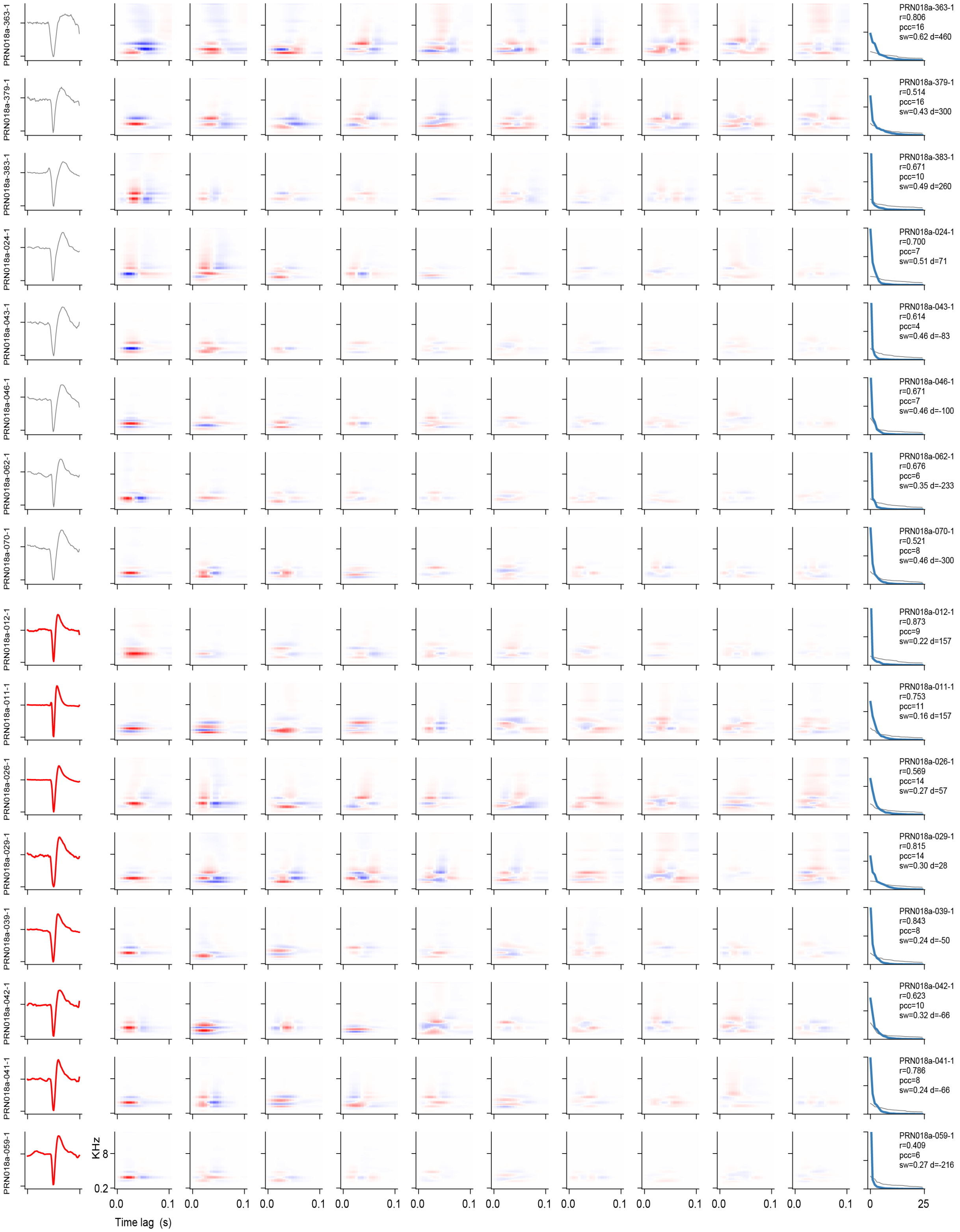

Figure S3. Ten highest-variance subspace filters for 16 single units recorded simultaneously from the same site (one unit per row). Left column shows mean spike waveform (gray: regular-spiking; red: narrow-spiking). Right column shows fraction dSTRF variance explained by each dimension. Numbers at left indicate prediction correlation (r), number of PCs required for 90% dSTRF variance (pcc), spike peak-trough width in ms (sw), and depth, um below the L3/4 boundary (d).

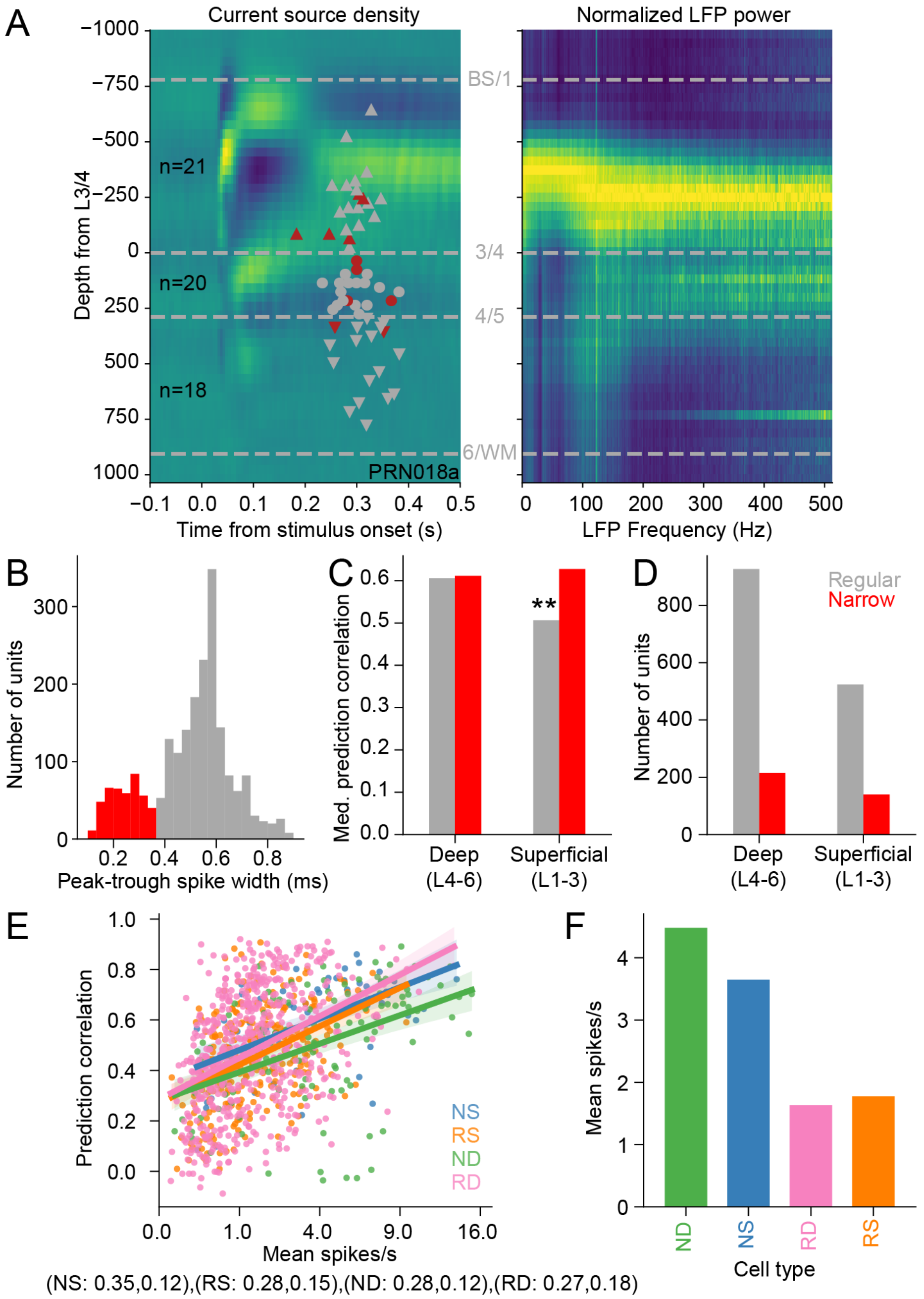

Figure S4. A. Current source density (CSD) evoked by narrowband noise, measured across leads of a Neuropixels probe spanning 2 mm of A1 (right). Gray/red shapes indicate depth of single regular-spiking/narrow-spiking neurons isolated during recording. Dashed lines indicate layer boundaries identified by CSD features. Local field potential (LFP) power spectrum computed across the same data, normalized to the maximum across depths. The band of high power provides a secondary indicator of L3. B. Histogram of spike widths, regular (R)/narrow (N) classes indicated by gray/red color (n=1751 sound responsive units). Neurons were also grouped by depth, superficial (S) above the L34 boundary and deep (D) below. **C.** Median CNN prediction correlation for each neuron group (RS vs. RD: **p==1.42e-13; comparisons for between all classes excluding RS: p>0.1, sign test). Number of units in each class. **E.** Scatter plot of mean spike rate vs. prediction correlation for each unit, colored by cell type. Numbers at bottom indicate intercept and slope of line fit to data from each group. **F.** Mean spike rate in each group.

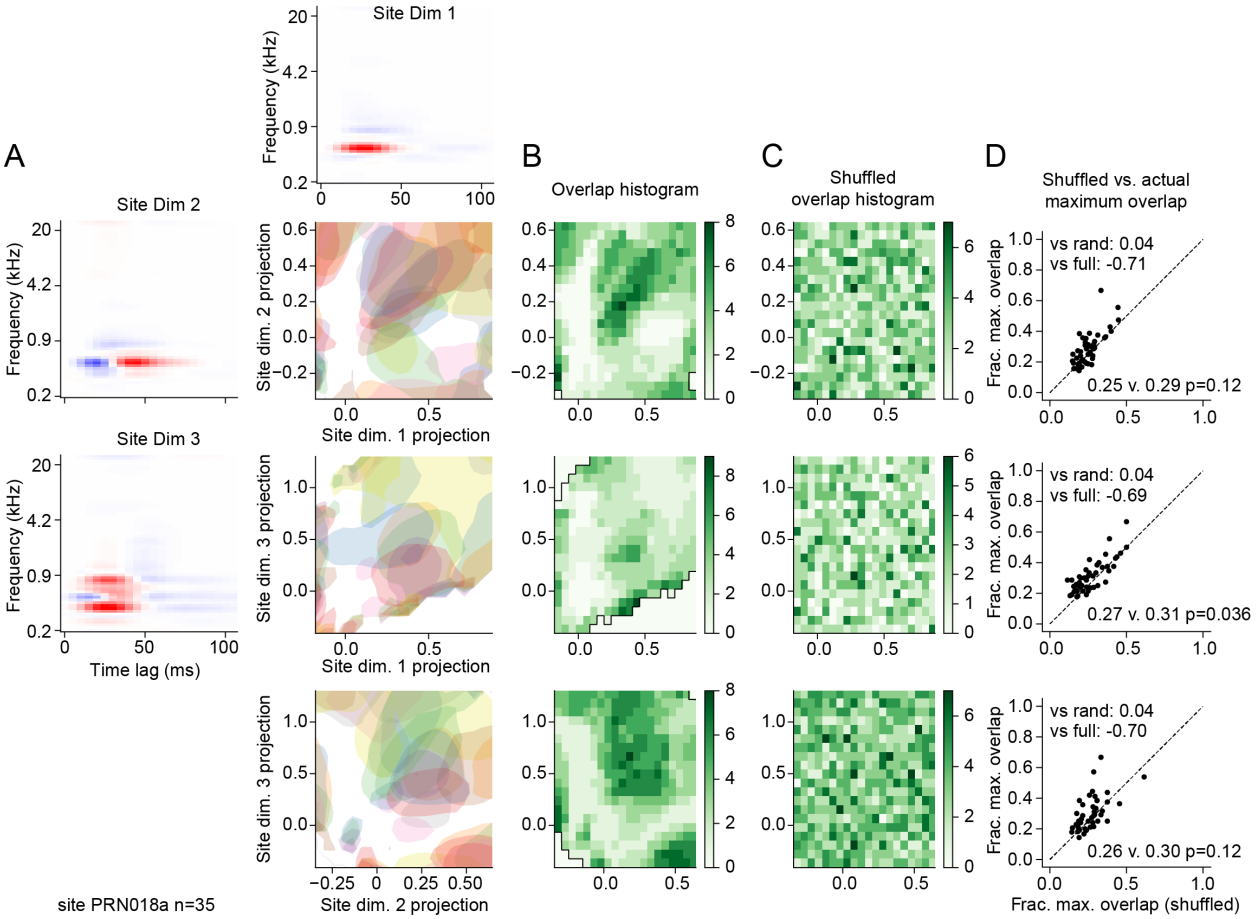

Figure S5. Detailed subspace tuning overlap for one recording site. **A.** Top three shared subspace filters (Site Dim 1-3) computed from aggregate dSTRF of 35 single units recorded across cortical layers in single site. Shaded plots show tuning space (contour of 80%-max response) of each unit in the shared space, projected onto pairs of subspace dimensions (top to bottom: 1 vs. 2, 1 vs. 3, 2 vs. 3). **B.** Two-dimensional histogram of overlap tuning spaces at each point in the stimulus subspace. **C.** Histogram of overlap after shuffling tuning spaces. **D.** Scatter plot shows fraction maximum overlap for actual vs. shuffled tuning spaces for each recording site (n=39 sites with >10 sound-responsive units). Numbers indicate mean difference in actual overlap from random shuffled and complete overlap (fraction=1.0), and p-values indicate significance of difference from random (Wilcoxon signed rank test).

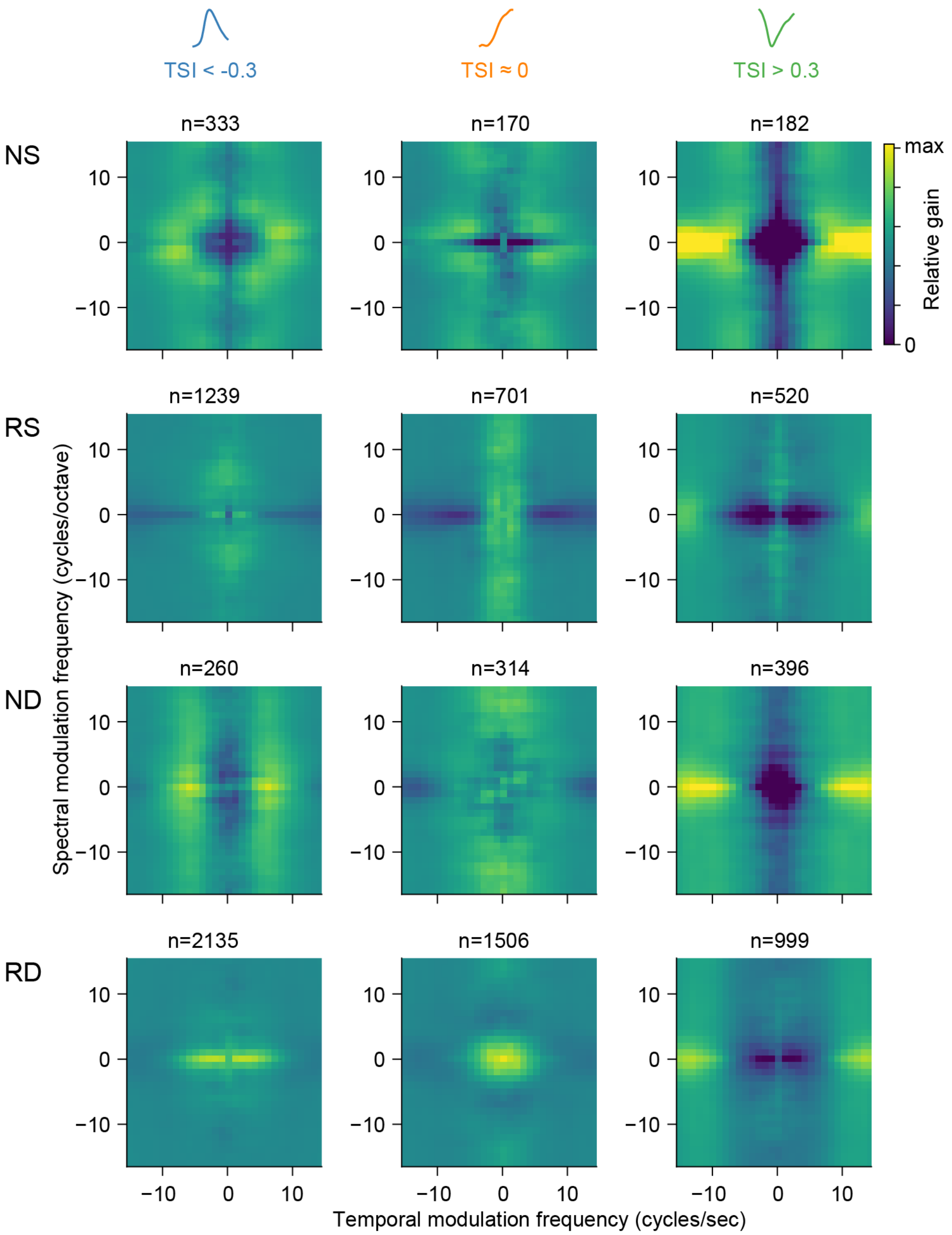

Figure S6. Mean spectro-temporal modulation tuning function for subspace filters grouped by cell type (row) and TSI value (columns). N indicates the total number of filters includes (across all significant filters for each unit).
